# Metal Nanoparticles with Antimicrobial Properties: The Toxicity Response in Mouse Mesenchymal Stem Cells

**DOI:** 10.3390/toxics11030253

**Published:** 2023-03-09

**Authors:** Pavel Rossner, Tereza Cervena, Barbora Echalar, Katerina Palacka, Alena Milcova, Zuzana Novakova, Michal Sima, Zuzana Simova, Jolana Vankova, Vladimir Holan

**Affiliations:** 1 Department of Nanotoxicology and Molecular Epidemiology, Institute of Experimental Medicine CAS, 142 00 Prague, Czech Republic; 2 Department of Genetic Toxicology and Epigenetics, Institute of Experimental Medicine CAS, 142 00 Prague, Czech Republic

**Keywords:** metal nanoparticles, antimicrobial properties, mesenchymal stem cells, toxicity

## Abstract

Some metal nanoparticles (NP) are characterized by antimicrobial properties with the potential to be used as alternative antibiotics. However, NP may negatively impact human organism, including mesenchymal stem cells (MSC), a cell population contributing to tissue growth and regeneration. To address these issues, we investigated the toxic effects of selected NP (Ag, ZnO, and CuO) in mouse MSC. MSC were treated with various doses of NP for 4 h, 24 h, and 48 h and multiple endpoints were analyzed. Reactive oxygen species were generated after 48 h CuO NP exposure. Lipid peroxidation was induced after 4 h and 24 h treatment, regardless of NP and/or tested dose. DNA fragmentation and oxidation induced by Ag NP showed dose responses for all the periods. For other NP, the effects were observed for shorter exposure times. The impact on the frequency of micronuclei was weak. All the tested NP increased the sensitivity of MSC to apoptosis. The cell cycle was most affected after 24 h, particularly for Ag NP treatment. In summary, the tested NP induced numerous adverse changes in MSC. These results should be taken into consideration when planning the use of NP in medical applications where MSC are involved.

## 1. Introduction

Nanoparticles (i.e., particles with at least one dimension < 100 nm) (NP), are widely used in many areas of human life [1]. Due to their specific properties, some NP have been suggested as potential antimicrobial agents with applications in medicine, e.g., for wound healing [2,3]. However, the administration of NP may potentially exert various negative biological effects, mostly stemming from the production of reactive oxygen species (ROS), that results in the damage of cell membranes and macromolecules, the disturbance of the cell cycle, or changes in the expression of numerous genes, including those involved in inflammatory response (reviewed, e.g., in [4]). Thus, the use of NP as an antimicrobial agent in medical applications may negatively affect stem cells that play a key role in the development of the organism and in cell differentiation, thus affecting the healing processes [5]. It is therefore important to identify any potential adverse impacts of NP on stem cells when they are used simultaneously for medical applications.

Stem cells, characterized by self-renewal and the ability to differentiate into other cell types, are classified into several groups [6,7]. (1) Embryonic stem cells (ESCs), prepared from blastocysts, can differentiate into numerous cell types, yet their therapeutic use might be problematic due to the ethical issues associated with their isolation and with teratoma formation after their transplantation. (2) Induced pluripotent stem cells (iPSCs), prepared from adult somatic cells by introducing the genes associated with stemness, can be efficiently differentiated into various distinct cell types. (3) Adult stem cells are found in nearly all the tissues of an adult organism, where they are often present as very small cell populations. They have the ability to differentiate not only into the cells of a particular tissue or organ but also into other cell types. Mesenchymal stem cells (MSC), which represent a special population of adult stem cells, have potent immunomodulatory and secretory properties [8,9,10]. They are a source of numerous cytokines and growth factors which contribute to tissue growth and regeneration. Essentially, they are considered the most prospective stem cell type for use in regenerative medicine.

In medical applications, metal NP are commonly used. These NP, made either of pure metals (e.g., Au, Ag, Ti, Zn) or their compounds, can serve as contrast materials for in vivo imaging, can be used for drug delivery, or applied as antitumor or antimicrobial agents [11]. Their antimicrobial properties make metal NP ideal candidates for alternative antibiotics, which may replace traditional drugs to which bacteria are becoming resistant. The mechanisms of the antibacterial activities of NP generally include membrane disruption, the production of ROS leading to oxidative stress, and the formation of coordination bonds between metal ions and N, O, and S atoms in biomolecules.

Several groups of metal NP have been considered as antimicrobial agents [11]. Silver NP (Ag NP) have become the best-known and the most commonly used antimicrobial NP. Ag NP are applied in wound dressings, as well as for the coating of medical devices and as carriers of chemotherapeutic drugs. They are active against both Gram-positive and Gram-negative bacteria. The biological effects of Ag NP can be attributed not only to direct NP-cell contact but also to Ag+ ions or soluble Ag+ complexes [12,13,14]. 

Although copper oxide NP (CuO NP) also possesses antibacterial properties [15,16,17], they have not yet been used for this purpose in medical practice. The mechanisms of their antimicrobial activities are not well understood; however, the role of ROS production is expected. Similar to Ag NP, they are active against both Gram-positive and Gram-negative bacteria. Several studies have linked the CuO NP cytotoxicity to free Cu^2+^ ions produced in the cells after NP internalization [18,19,20,21,22]. Other mechanisms may include direct interaction with the mitochondrial membranes, inducing apoptosis and autophagy response [23,24,25]. 

Zinc oxide NP (ZnO NP) are more effective against Gram-positive bacteria, most likely due to the mechanisms of their interactions with bacterial cell walls, in which the lack of a lipopolysaccharide layer plays a role. The proposed antimicrobial mechanism is via visible light irradiation and membrane damage, as well as ROS production and the release of Zn^2+^ ions [26]. ZnO NP also have pro-inflammatory properties manifested by the induction of cytokine expression, and they also show promise for cancer therapy. As with CuO NP, ZnO NP are not routinely clinically used.

The application of standard antibiotics accelerates the healing of cutaneous wounds, most likely via the mechanisms involving decreased inflammation and an increased number of fibroblasts, extracellular matrix components, and re-epithelization [27]. Similarly, the therapeutic potential of MSC might be potentiated by the simultaneous application of antimicrobial NP (e.g., metal NP). However, the toxicity of these NP may limit their wider medical use.

The rationale of our study was to identify the mechanisms of metal NP toxic response in MSC, as well as to characterize the physicochemical properties of these NP in relevant incubation conditions. We focused on a comparison of three NP with antimicrobial properties and conducted a comprehensive set of toxicity tests. In contrast to previous reports, this design allowed us to obtain reliable information on differences in the biological impacts of metal NP in MSC and their potential to be used in a combined treatment with stem cells. Specifically, we analyzed the impact of Ag, ZnO, and CuO NP on mice MSC after 4 h, 24 h, and 48 h of exposure. We assessed a panel of toxicity parameters including oxidative stress-related markers (ROS production, lipid peroxidation, DNA oxidation), DNA breaks, the induction of apoptosis, and cell cycle alterations. In order to elucidate the NP behavior in our exposure setup, we analyzed dynamic light scattering (DLS) and confirmed cell internalization by transmission electron microscopy (TEM). While our data showed a complex response that depends on the type of NP tested and the exposure conditions, we generally detected the toxicity of NP in MSC. ZnO NP was identified as comparatively the least toxic in our experimental conditions. Overall, we suggest that the application of the tested NP might not be optimal for combined wound treatment with MSC. This conclusion, however, should be confirmed in additional experiments, particularly in in vivo animal tests.

## 2. Materials and Methods

### 2.1. MSC Cell Culture

In all experiments, female BALB/c mice at the age of 10–16 weeks were used. The animals were obtained from the Institute of Molecular Genetics of the Czech Academy of Sciences, Prague. The use of animals was approved by the local Animal Ethics Committee of the Institute of Experimental Medicine of the Czech Academy of Science, Prague.

In order to obtain a high enough cell count, we isolated MSCs from inguinal fat pads. Small cut pieces were digested for 60 min at 37 °C with a 1% solution of collagenase I (Sigma-Aldrich, St. Louis, MO, USA) in Hanks’ balanced salt solution with Ca^2+^ and Mg^2+^. The cell suspension was then twice centrifuged (250 g, 8 min) in a complete medium (DMEM + 10% FBS + 100 U/mL of penicillin + 100 µg/mL of streptomycin + 10 mM HEPES buffer). The cell pellets were resuspended and seeded in T75 cell culture flasks. After 48 h cultivation, the non-adherent cells were washed out and the adherent cells were continuously cultured for 14 days with regular maintenance. In all the experiments, the cells harvested in the 3rd to 4th passage were used.

MSC were adherent to plastic surfaces and had a typical fibroblast-like morphology [28]. Phenotypic characterization of MSC was carried out using LSRII flow cytometer (BD Biosciences, Franklin Lakes, NJ, USA), and analyzed using FlowJo 10 software (Tree Star, Ashland, OR, USA). Briefly, MSC were washed in a phosphate-buffered saline (PBS), and incubated for 30 min at 4 °C with anti-mouse monoclonal antibodies: allophycocyanin (APC)-labeled anti-CD44 (clone IM7, BD PharMingen, San Jose, CA, USA), phycoerythrin (PE)-labeled anti-CD105 (clone TY/11.8, eBioscience, San Diego, CA, USA), fluorescein isothiocyanate (FITC)-labeled anti-CD90.2 (clone 30-H12, BioLegend, San Diego, CA, USA), FITC-labeled anti-CD45 (clone 30-F11, BioLegend, San Diego, CA, USA), APC-labeled anti-CD11b (clone M1/70, BioLegend, San Diego, CA, USA) and PE-labeled anti-CD31 (clone MEC 13.3, BD PharMingen, San Jose, CA, USA). As negative controls, cells stained with PE-labeled rat IgG2a (clone RTK2758, BioLegend, San Diego, CA, USA), APC-labeled rat IgG2b (clone RTK4530, BioLegend, San Diego, CA, USA) or FITC-labeled rat IgG2b (clone eB149/10H5, eBioscience, San Diego, CA, USA) were used, and Hoechst 33258 fluorescent dye (Invitrogen, Carlsbad, CA, USA) was used to distinguish dead cells. Detailed phenotypic data were reported previously [29,30].

### 2.2. Concentration Selection and Nanoparticle Characterization

#### 2.2.1. WST-1 and Exposure Scheme

All concentrations used in this study were chosen based on the cytotoxicity assay WST-1 (Roche, Mannheim, Germany), measuring cell proliferation and viability. Briefly, MSC in a concentration of 1 × 10^4^ cells/mL were seeded in 96-well tissue culture plates (Nunc, Roskilde, Denmark). After 24 h of cultivation, Ag, ZnO, CuO NP at concentrations ranging from 0.2 to 50 µg/mL were added for the next 4, 24, or 48 h, and the assay was performed according to the manufacturer’s instructions (see [29] for details).

In all subsequent experiments, MSC were exposed to the following non-cytotoxic/subtoxic concentrations of NP based on the results of the WST-1 assay: Ag NP: 1.5, 3.12, 6.25 µg/mL; ZnO NP: 0.75, 1.5, 3.12 µg/mL; and CuO NP: 0.2, 0.3, 0.4 µg/mL, for 4, 24 and 48 h. All tested parameters were carried out in biological triplicate (technical hexaplicate) in two independent experiments.

#### 2.2.2. Sample Preparation and Dynamic Light Scattering (DLS)

In this project, three types of metal nanoparticles were chosen: Ag (uncoated silver nanopowder, <150 nm particle size, Sigma-Aldrich, St. Louis, MO, USA), ZnO (uncoated NM110; 70–90 nm particle size, JRC Nanomaterials Repository) and CuO (copper oxide nanopowder, <50 nm particle size, Sigma-Aldrich, St. Louis, MO, USA) (representative transmission electron microscopy (TEM) images are shown in Supplementary Figure S1). Nanoparticle suspension was freshly prepared before every experiment according to the NANOREG suspension protocol [31]. Briefly, 15.36 mg of NP were pre-wetted with 30 µL of 95% ethanol and diluted in 5.97 mL deionized water (diH_2_O) containing 0.05% bovine serum albumin (BSA, Sigma-Aldrich, St. Louis, MO, USA), to a total concentration of 2.56 mg/mL (batch). Batches were sonicated for 16 min in an ice bath (400 W, 10% amplitude, 3136 MJ/m^3^) by an ultrasonic homogenizer (S-450d, Branson, Brookfield, CT, USA). Exposure concentration was obtained by diluting a batch in the complete cell culture medium to a final concentration of 1.5, 3.125, 6.25 µg/mL for Ag NP; 0.75, 1.5, 3.125 µg/mL for ZnO NP and 0.2, 0.3, 0.4 µg/mL for CuO NP.

Each NP type and concentration dilution was characterized using a DLS analysis (Zetasizer Nano ZS, Malvern, UK) at T0 (immediately after preparation), T4 (4 h after preparation), T24 (24 h after preparation) and T48 (48 h after preparation). In the interval between T0 and T4, T24 and T48, the samples were kept in a cell incubator (5% CO_2_, 37 °C, and high relative humidity) to mimic cell exposure conditions. DLS was measured in polystyrene cell cuvettes (DTS0012) with a stabilization time of 120 s, at 25 °C (batch) and 37 °C (medium suspensions), and each sample had 12–24 repeated runs.

#### 2.2.3. Transmission Electron Microscopy

Nanoparticle internalization in the cells was verified by TEM. Mouse mesenchymal stem cells were incubated with Ag, ZnO, and CuO nanoparticles for 24 h (at a concentration of 6.25 µg/mL for Ag NP, 3.125 µg/mL for ZnO NP, 0.4 µg/mL for CuO NP), fixed with 2.5% glutaraldehyde in 0.1 M Sorensen’s buffer (7:3; Na_2_HPO_4_ × 12 H_2_O: KH_2_PO_4_), stained with 1% osmium tetroxide in 0.1 M Sorensen’s buffer for 2 h and by 1% ethanolic solution of uranyl acetate overnight (in the dark). The samples were then dehydrated in ethanol, immersed in propylene oxide, and flat embedded in Epoxy resin (Epon-Durcupan) using gelatin capsules. After polymerization for 72 h at 60 °C, the coverslips were removed using liquid nitrogen. Ultrathin sections of 60 nm were cut with Ultracut S ultramicrotome (Reichert/Leica, Wetzlar, Germany) equipped with a diamond knife (45°; Diatome), placed on formvar-coated cooper grids, and examined in an FEI Morgagni 268(D) transmission electron microscope (Philips/FEI, Hillsboro, OR, USA) at 80 kV. Images were captured with a MegaView II CCD camera (Olympus Corp., Münster, Germany).

### 2.3. ROS Production and Lipid Peroxidation

Intracellular ROS production was measured using a ROS/Superoxide Detection assay kit (ab139476, Abcam, Cambridge, UK) according to the manufacturer’s instructions. Briefly, MSC grown in 96 well black wall/clear bottom plates (Corning^®^ 96-well plates, Sigma Aldrich, St. Louis, MO, USA) were incubated with selected concentrations of NP for 4, 24, and 48 h in the presence of the ROS/Superoxide Detection Solution. Pyocyanin (100 µM) was used as a positive control and 0.1 µL/mL DMF (Sigma Aldrich, St. Louis, MO, USA) as vehicle control. ROS production (Ex = 488 nm, Em = 520 nm) and superoxide production (Ex = 550 nm, Em = 610 nm) was calculated as relative fluorescence intensity normalized to the fluorescence of the negative control, using SpectraMax iD3 (Molecular Devices, San Jose, CA, USA). 

15-F_2t_-isoprostane concentration was measured in MCS lysates using an 8-isoprostane ELISA kit (Cayman Chemicals Company, Ann Arbor, MI, USA) according to the manufacturer’s protocol, with some modifications as previously described [32,33]. The total protein concentration was detected using a Bicinchoninic Acid kit (Sigma–Aldrich, St. Louis, MO, USA), and 15-F2t-IsoP was assessed in samples containing 100 µg of total proteins. The mean 15-F2t-IsoP level of the exposed cells was compared with the negative controls (the cells incubated in culture media), and the differences between the groups were evaluated using *t*-test.

### 2.4. DNA Alterations

#### 2.4.1. Comet Assay

A modified protocol for alkaline Comet assay (detecting both strand breaks and oxidized purines) with and without bacterial repair enzyme DNA-formamidopyrimidine glycosylase (Fpg; M0240S, NEB, Ipswich, MA, USA), was used [34]. A suspension of MSC harvested at T4, T24 and T48 was frozen (0.6–1.0 × 10^6^ cells/mL) upon further processing. For the assay, 20 µL from the cell suspension was mixed with low-melting-point agarose and dropped onto slides pre-covered with normal-melting-point agarose. The slides were incubated for 1 h in lysis solution (2.5 M NaCl, 100 mM EDTA, 10 mM Tris, 0.16 M DMSO, 0.016 mM Triton X-100, pH 10) at 4 °C. After incubation, the slides were washed with PBS and treated with Fpg or buffer used for the enzyme dilution (0.1 M KCl, 4 mM EDTA, 2.5 mM HEPES, 2% BSA) for 30 min at 37 °C. DNA unwinding was performed for 40 min in alkaline buffer (0.3 M NaOH, 1 mM EDTA, pH 13). Fresh alkaline buffer was used for electrophoresis (30 min, 1.2 V/cm, 300 mA). After electrophoresis, the slides were neutralized in 0.4 M Tris (pH 7.5) and stained with SYBR™ Safe DNA Gel Stain (Invitrogen, Waltham, MA, USA). The analysis was carried out using the Metafer MF-1273 system with H-3060-055-NA Lamp Module for X-Cite Exacte (MetaSystems, Heidelberg, Germany), and the results were expressed as a percentage of DNA in the tail (tail DNA%). The level of oxidative DNA damage was calculated as the difference between the median of FPG-treated samples (total DNA damage) and buffer-treated samples (DNA strand breaks). The total DNA damage was measured in 100 randomly selected cells per slide, and the differences between the groups were analyzed using unpaired two-tailed *t*-test.

#### 2.4.2. Micronucleus Assay

The potential genotoxicity of the tested NP was measured in mononucleated cells (MNC) using the micronucleus test. At the end of cultivation (T4, T24, T48), the cells were trypsinized (Trypsin-EDTA 0.25%, Gibco, NY, USA), treated with a hypotonic solution of KCl (0.075 M, Sigma-Aldrich, St. Louis, MO, USA) and fixed with a mixture of methanol (Merck Millipore, Burlington, MA, USA) and acetic acid (Penta, Prague, Czech Republic) (3:1). At least two slides per sample were prepared, and dried slides were stained by 5% Giemsa (Merck Millipore). A total of 2000 mononucleated cells per tested compound were evaluated by visual scoring (Olympus BX41 microscope). The results are expressed as a relative value of micronuclei in 1000 mononucleated cells compared with the controls.

### 2.5. Cell Cycle Alterations and Apoptosis 

#### 2.5.1. Cell Cycle

MSC treated with NP were harvested at the end of cultivation (T4, T24, T48), and fixed with 70% ethanol overnight at 4 °C. The next day, the cells were washed in PBS and stained with the PI staining solution containing 1×Perm/Wash Buffer (BD Biosciences; San Jose, CA, USA), 50 µg/mL Ribonuclease A (Sigma-Aldrich, St. Louis, MO, USA), and 50 µg/mL Propidium Iodide Sigma-Aldrich, St. Louis, MO, USA) for 1 h at 4 °C. A cell cycle analysis of all the samples was then performed on the BD LSRII flow cytometer (BD Biosciences; San Jose, CA, USA). Due to PI staining, the samples were excited with 25 mW, 561 nm laser line, and fluorescence collected at 610/20 nm band. The data were analyzed with FlowJo 9.8.2 (BD Biosciences; San Jose, CA, USA) for Mac, using Watson cell cycle fitting algorithm, with manual sub G1 phase gating of singlet cells. 

#### 2.5.2. Gene Expression *Bax*/*Bcl-2*

The expression of *Bax* and *Bcl-2* was analyzed by real-time polymerase chain reaction (RT-PCR) using primers obtained from Generi Biotech (Hradec Kralove, Czech Republic; sequences shown in Table 1). The total RNA was extracted from MSC by TRI reagent (Molecular Research Centre, Cincinnati, OH, USA) according to the manufacturer’s instructions. One µg of RNA was mixed with deoxyribonuclease I (DNase I; Promega, Madison, WI, USA) in a DNase I buffer (Promega, Madison, WI, USA), and used for reverse transcription. The first cDNA strand was synthesized by random primers (Promega) and M-MLV reverse transcriptase (Promega, Madison, WI, USA) in a total volume of 25 µL. RT-PCR was performed using Power SYBR Green PCR master MIX (Applied Biosystems, Carlsbad, CA, USA) on a cycler StepOne Plus RT-PCR System (Applied Biosystems, Carlsbad, CA, USA). RT-PCR parameters included denaturation at 95 °C for 3 min, 40 cycles at 95 °C for 20 s, annealing at 60 °C for 30 s, and elongation at 72 °C for 30 s. Fluorescence data were collected at each cycle after an elongation step at 80 °C for 5 s. Data were analyzed using StepOne Software 2.3 (Applied Biosystems, Carlsbad, CA, USA), and the expression levels of the analyzed genes were normalized to those of the reference gene glyceraldehyde-3-phosphate dehydrogenase (*GAPDH*) using the 2^−∆∆Ct^ method [35].

## 3. Results

### 3.1. The Characterization of Nanoparticles and the Identification of Optimal Test Doses of NP (WST-1, DLS, TEM)

#### 3.1.1. WST-1

The selection of exposure concentrations was carried out using the cytotoxicity assay WST-1 (Roche, Mannheim, Germany), measuring cell proliferation and viability. Ag, ZnO, CuO NP at concentrations ranging from 0.2 to 50 µg/mL were added to MSC for 4 and 48 h (reported in [29]; significant changes in cytotoxicity were found for doses above 25 µg/mL, 12.5 µg/mL, and 0.2 µg/mL, for Ag, ZnO and CuO NP, respectively). Based on the results, the following concentrations were chosen for further testing: Ag NP (1.5, 3.12, 6.25 µg/mL), ZnO NP (0.75, 1.5, 3.12 µg/mL), and CuO NP (0.2, 0.3, 0.4 µg/mL).

#### 3.1.2. DLS

Selected concentrations of NP with low cytotoxicity were characterized by DLS using a Malvern Zetasizer Nano ZS (Table 2, Table 3 and Table 4). The average hydrodynamic size (z-average, nm) and the polydispersity index (PDI) value were obtained immediately after the preparation of NP dispersions, (T0), 4 h (T4), 24 h (T24) and 48 h (T48) after preparation. The consistent time-dependent z-average changes were observed for CuO NP and ZnO NP in all three concentrations (Table 3 and Table 4). In both cases, we observed a smaller hydrodynamic diameter of the dispersed NP and smaller PDI than that of the NP batch, regardless of the NP concentration. These results suggest that both types of NP showed signs of dissolving in the cell culture medium over time. On the other hand, for Ag NP, the average hydrodynamic diameter decreased by 1.5 and 3.125 µg/mL but increased for the highest concentration of 6.25 µg/mL. The PDI of Ag NP remained similar from T0 to T48 (1.5 µg/mL) or increased (3.125 and 6.25 µg/mL). According to the manufacturer [36], the PDI of measured NP should be <0.7 in order to obtain reliable results. This value exceeded the limit in some cases (indicated in red in Table 2 and Table 4).

#### 3.1.3. TEM

**Figure 1:**
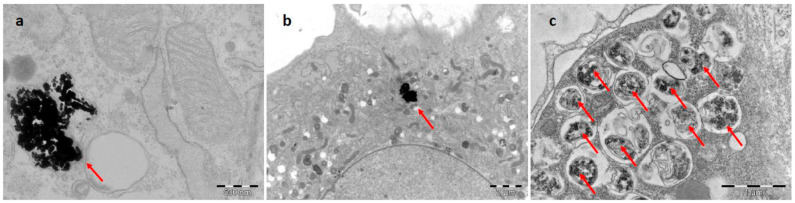
Transmission electron microscopy images of Ag (**a**), ZnO (**b**), and CuO (**c**) nanoparticles inside MSC. Mouse mesenchymal stem cells were incubated with Ag, CuO, and ZnO nanoparticles for 24 h (at a concentration of 6.25 µg/mL for Ag NP, 0.4 µg/mL for CuO NP and 3.125 µg/mL for ZnO NP). NP aggregates are shown by red arrows.

MSC were incubated with the highest selected concentration of each NP for 24 h and the internalization of NP in MSC was confirmed, as shown in Figure 1.

### 3.2. Oxidative Stress-Related Response

#### 3.2.1. ROS Production

**Figure 2:**
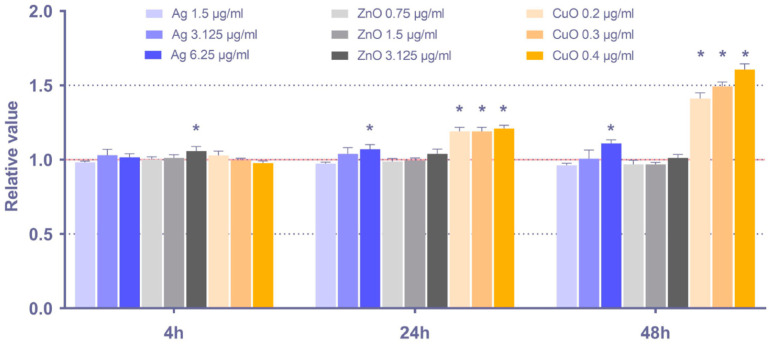
Relative intracellular ROS levels, calculated as ratio between the exposed and control samples, in MSC after 4 h, 24 h, and 48 h exposure to selected doses of Ag, ZnO, and CuO NP. The average values from six independent experiments are presented. The red line indicates the reference ROS levels in the controls. The asterisk denotes significant changes when compared with the untreated controls (*p* < 0.05).

The production of ROS is a prerequisite for oxidative stress induction and the subsequent damage of macromolecules. To elucidate the effect of the tested NP on ROS generation, intracellular ROS levels in MSC upon 4 h, 24 h, and 48 h exposure were analyzed (Figure 2; data for shorter exposure periods are shown in Supplementary Figure S2). Overall, the effects were relatively modest. CuO NP had the greatest potential to induce ROS production, as observed after 24 h and 48 h exposure. The effects of Ag and ZnO NP were comparatively weak and mostly limited to the highest tested concentrations of these NP. As the production of ROS might be induced at time periods shorter than 4 h, the ROS levels after 15 min, 30 min, 1 h, and 2 h exposure were checked, but no consistent significant increase was observed (data not shown).

#### 3.2.2. Oxidative DNA Damage

**Figure 3:**
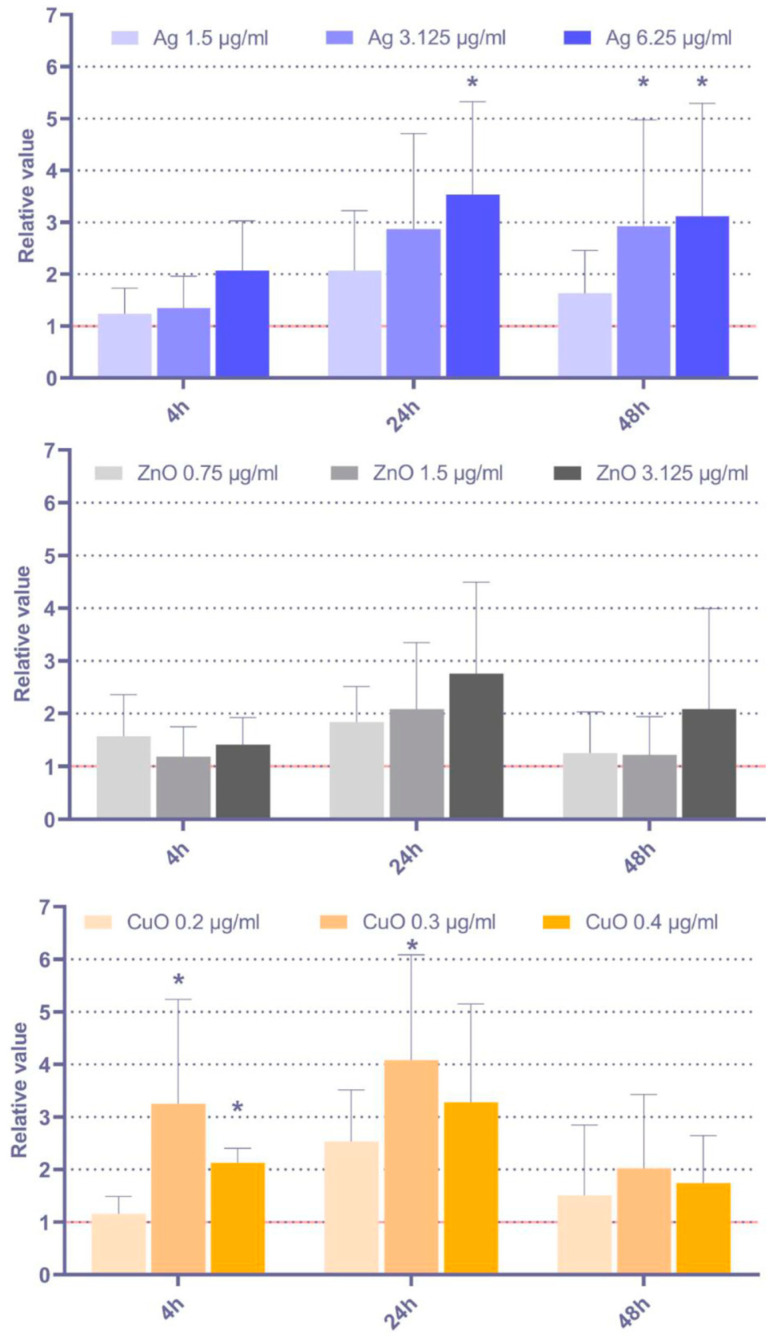
Induction of DNA oxidation in MSC (relative values, calculated as ratio between the exposed and control samples) after 4 h, 24 h, and 48 h exposure to selected doses of Ag, ZnO, and CuO NP. The average values from six independent experiments are presented. The red line indicates the reference DNA oxidation in the controls. The asterisk denotes significant changes when compared with the untreated controls (*p* < 0.05).

DNA oxidation was analyzed using the Comet assay based on the presence of the Fpg-sensitive sites. Overall, a relatively weak response was detected for all the NP, with significant results observed after exposure to Ag NP (the 24 h and 48 h treatment) and CuO NP (the 4 h and 24 h incubation). For the Ag NP exposure, a dose response was found. Interestingly, no significant effects were observed after the ZnO NP exposure (Figure 3).

#### 3.2.3. Lipid Peroxidation

**Figure 4:**
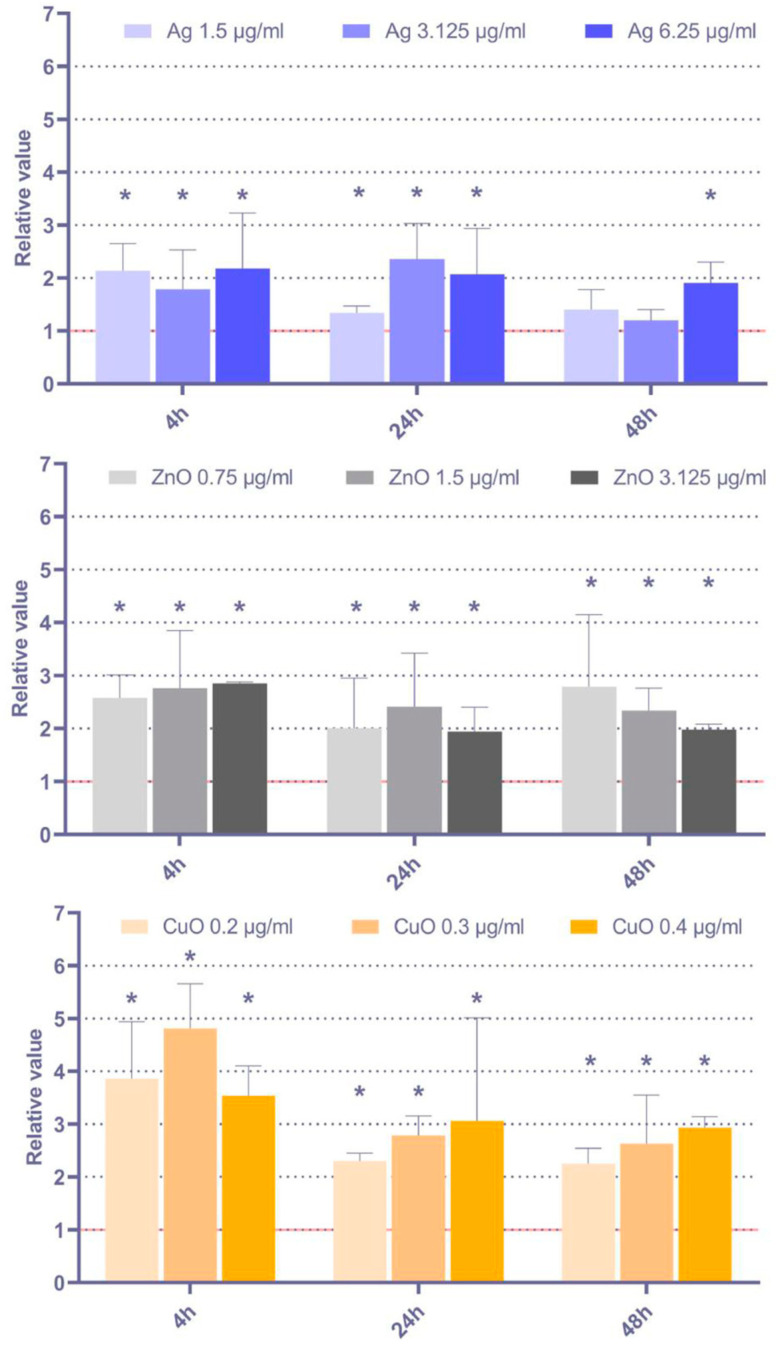
Relative IsoP levels, calculated as ratio between the exposed and control samples, after 4 h, 24 h, and 48 h exposure to selected doses of Ag, ZnO, and CuO NP. The average values from six independent experiments are presented. The red line indicates the reference IsoP levels in the controls. The asterisk denotes significant changes when compared with the untreated controls (*p* < 0.05).

Lipid peroxidation was assessed as the levels of IsoP, a generally accepted marker of ROS impact on lipids. The analysis of intracellular and membrane-bound IsoP showed elevated lipid peroxidation after exposure to all tested NP at most of the time intervals (Figure 4). The most pronounced effects were detected upon CuO NP exposure, particularly after the 4 h treatment, while the effects of Ag NP were relatively weak with no significant response after treatment with 1.5 and 3.125 µg/mL for 48 h.

### 3.3. DNA Damage

#### 3.3.1. The Induction of DNA Strand Breaks

**Figure 5:**
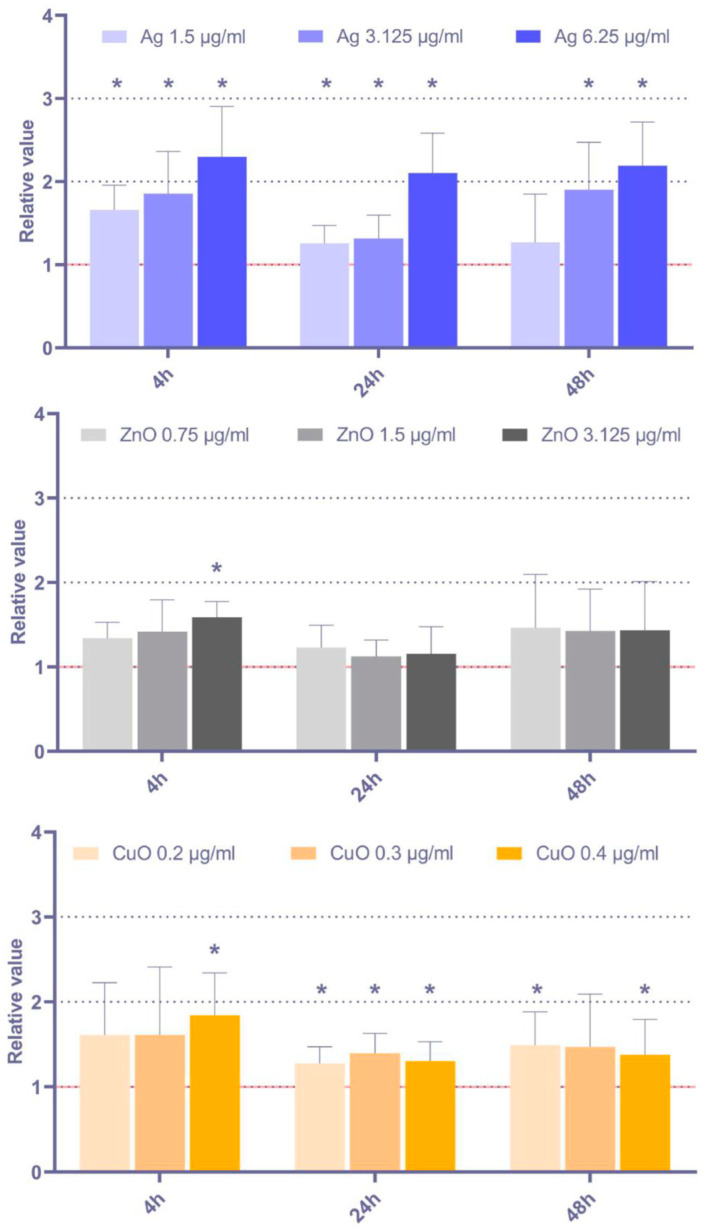
DNA breaks (relative levels, calculated as ratio between the exposed and control samples,) after exposure of MSC to Ag, ZnO, and CuO NP at selected doses for 4 h, 24 h, and 48 h. The average values from six independent experiments are presented. The red line indicates the reference levels of DNA breaks in the controls. The asterisk denotes significant changes when compared with the untreated controls (*p* < 0.05).

The Comet assay was used to detect single- and double-strand DNA breaks. Ag NP were the most potent inducer of DNA fragmentation—a significant increase was detected for all but one of the exposure conditions, and a dose response was observed. The effects of CuO NP treatment were mostly limited to longer exposure periods (24 h and 48 h). ZnO NP had a minimal impact on the induction of DNA breaks (Figure 5).

#### 3.3.2. Micronuclei Formation

**Figure 6:**
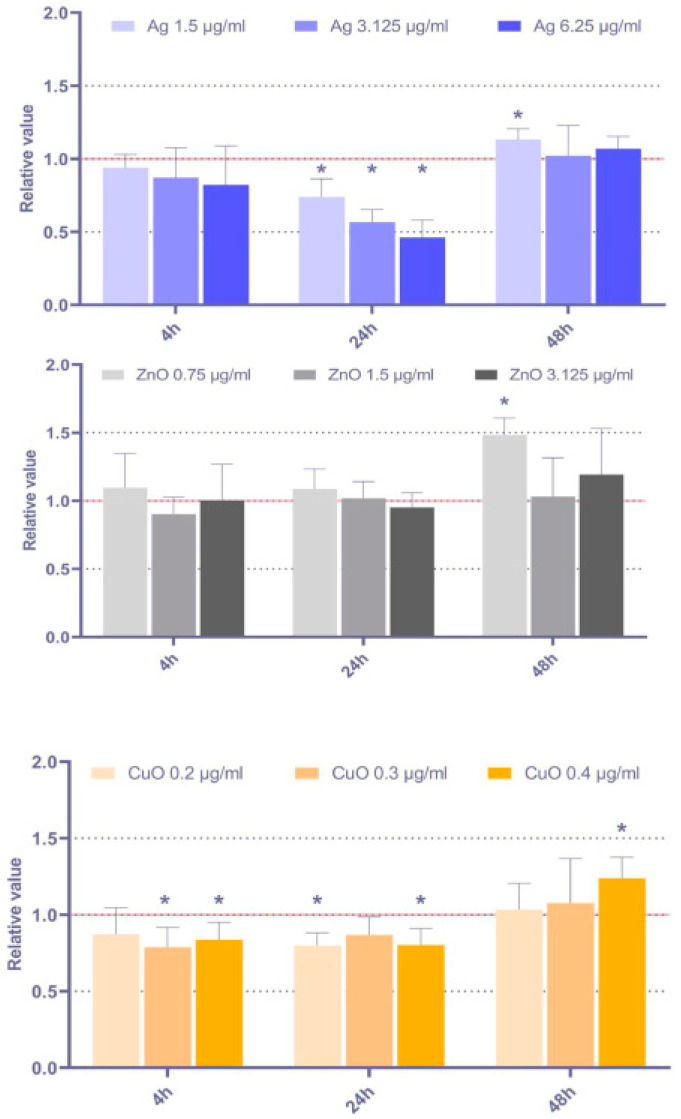
Relative frequency of micronuclei, calculated as ratio between the exposed and control samples, after 4 h, 24 h, and 48 h exposure to selected doses of Ag, ZnO, and CuO NP. The average values from six independent experiments are presented. The red line indicates the reference frequency of MN in the controls. The asterisk denotes significant changes when compared with the untreated controls (*p* < 0.05).

The treatment of MSC with metal NP had a minimal impact on the induction of the frequency of micronuclei: for all the NP, increased levels were detected only after the 48 h exposure period for a limited number of doses. Interestingly, both Ag and CuO NP caused a decrease in MN frequency after 4 h and 24 h of exposure (Figure 6).

### 3.4. Cell Cycle Alterations and Apoptosis

#### 3.4.1. Cell Cycle Analysis

**Figure 7:**
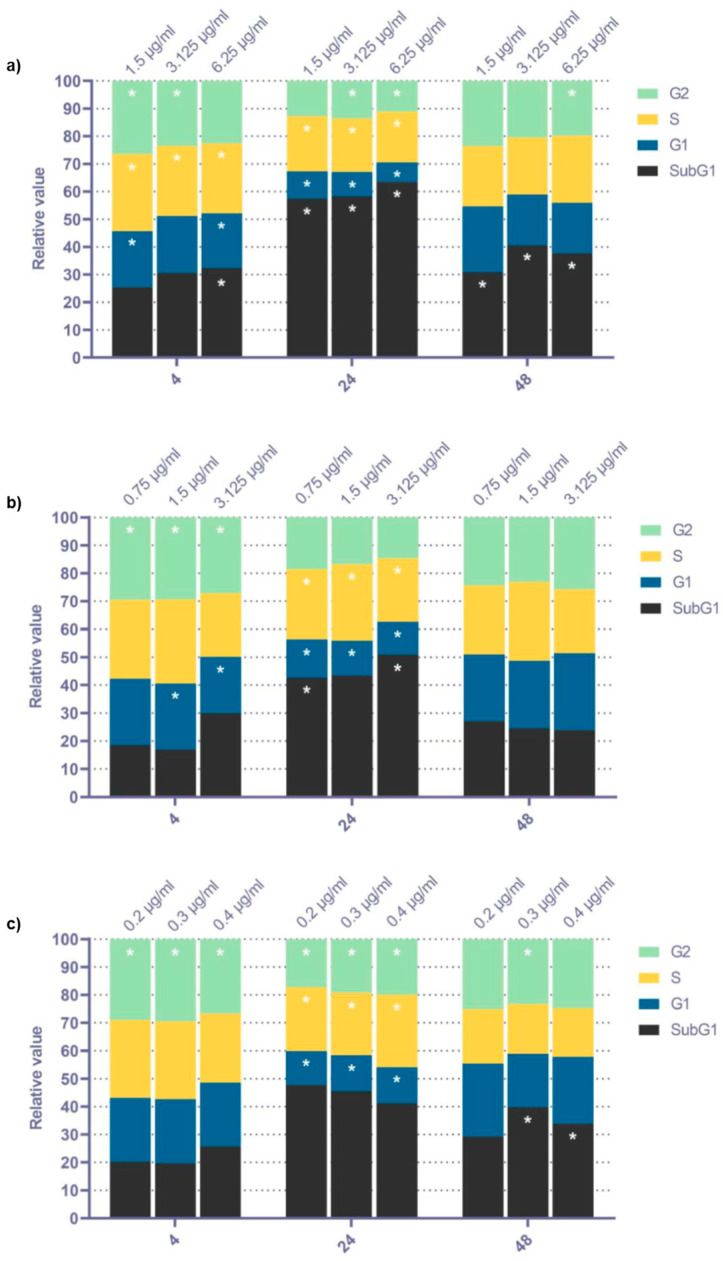
Distribution of cell cycle phases (relative proportions, calculated as ratio between the exposed and control samples) after 4 h, 24 h, and 48 h exposure to selected doses of Ag (**a**), ZnO (**b**), and CuO NP (**c**). The average values from six independent experiments are presented. The asterisk denotes significant changes when compared with the untreated controls (*p* < 0.05).

The distribution of cell cycle phases of MSC after exposure to metal NP is reported in Figure 7. The most pronounced effects were detected for the Ag NP exposure, followed by CuO NP and ZnO NP. The 24 h Ag NP treatment increased the proportion of cells in the sub-G1 phase, which corresponds to apoptotic cells. This exposure simultaneously decreased the proportion of G1 and G2 phase cells. This trend, albeit less pronounced, was also detected for ZnO and CuO NP. The impact of the 4 h and 24 h NP exposure on cell cycle distribution was less and, specifically for the 48 h ZnO NP exposure, without any significant alterations.

#### 3.4.2. Sensitivity to Apoptosis Induction

**Figure 8:**
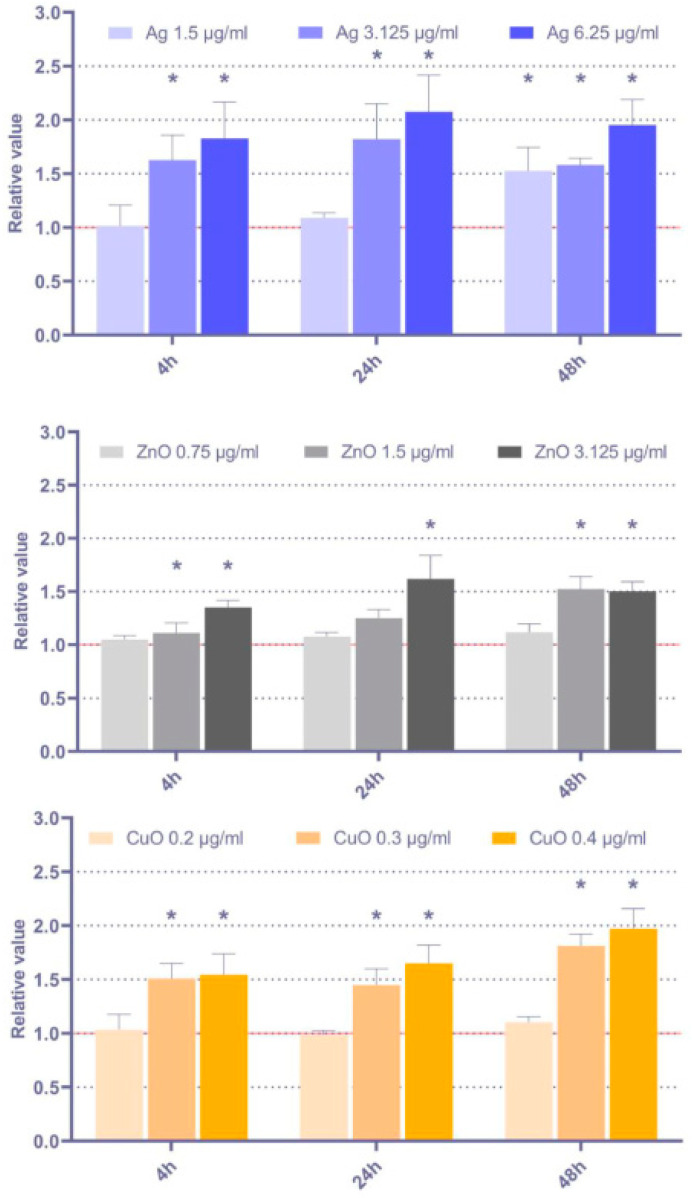
Apoptosis induction (relative values of *Bax*/Bcl2 gene expression) after 4 h, 24 h, and 48 h exposure to selected doses of Ag, ZnO, and CuO NP. The average values from three independent experiments are presented. The red line indicates the reference relative expression of both genes in the controls. The asterisk denotes significant changes when compared with the untreated controls (*p* < 0.05).

The effect of the tested NP on MSC sensitivity to apoptosis induction was assessed based on the analysis of the expression of the pro-apoptotic *Bax* gene and the anti-apoptotic *Bcl-2* gene. As indicated in Figure 8, a significant pro-apoptotic response was detected for all the tested NP and time periods, although in most cases, the lowest tested dose had no impact. While the response is mostly comparable across the tested NP, the effects induced by Ag NP seemed to be the most pronounced, while the impact of ZnO NP was the least significant one.

## 4. Discussion

Mesenchymal stem cells represent a cell subpopulation with potent immunomodulatory and secretory properties that are a source of numerous cytokines and growth factors. These molecules, along with exosomes produced by MSC, are believed to be key factors that support the healing processes in which MSC are involved [23,24,37]. Metal nanoparticles as antimicrobial agents can further facilitate wound healing [25]. However, NP may also cause damage to macromolecules, which may negatively affect the function of MSC and, in turn, the healing process itself. Gathering sufficient information on the mechanisms of the biological response of MSC upon their contact with NP is a key step before the combination of MSC and metal NP can be considered as an approach to support wound healing in medical applications.

The biological effects of NP exposure are linked to two major mechanisms: (1) direct interactions of NP with macromolecules and (2) indirect impacts of ROS and toxic ions (for soluble NP) on cellular systems. The direct interactions may affect DNA replication and transcription or cell division by physically interfering with chromosome separation, as well as with the proteins involved, e.g., in cell cycle regulation, intracellular signaling, regulation of gene expression, DNA replication, transcription, translation, or mitosis [38]. ROS formation is induced by NP either due to the presence of transition metals or by the interference of NP with mitochondria. ROS cause oxidative damage to macromolecules (DNA, lipids, and proteins), affecting their functions, damaging their structure (e.g., causing DNA breaks), and potentially leading to the induction of mutations [39]. As secondary messengers, they may change gene expression, thus impacting cell proliferation and differentiation.

In our study, we aimed to investigate the biological impacts of selected metal NP with known antimicrobial activity on MSC, to evaluate their suitability for a combined (MSC + NP) method of wound treatment. These tests are a prerequisite for subsequent experiments in which the benefits of the administration of NP along with MSC for wound healing will be verified. The studied NP included those that have already been established for use in medical applications (Ag NP), as well as antimicrobial NP that have not been reported as agents tested in medicine (ZnO NP, CuO NP).

The studies of Ag NP effects on MSC showed contradictory results. Sengstock et al., observed a significant concentration-dependent decrease in human MSC adipogenic and osteogenic ability, but no effect on chondrogenic differentiation following Ag NP exposure [40]. Hackenberg et al. reported DNA damage detected by the Comet assay, even at the non-cytotoxic concentration of 0.1 µg/mL Ag NP after 1 h of treatment [41]. Interestingly, decreased MSC migration was only detected when a cytotoxic concentration of 10 µg/mL of Ag NP was used. In contrast, some studies in human and mouse MSC have shown a positive influence of Ag NP on MSC differentiation, even though the uptake of Ag NP was confirmed [42,43]. Similar results were obtained when the effects of scaffolds, dressings, or materials used for implants containing Ag NP, were investigated [44,45,46,47]. The inconsistency in the results is probably related to differences in experimental systems used in the studies—the origin of stem cells, exposure conditions, and physicochemical properties of NP.

The studies focused on the investigation of CuO NP and ZnO NP toxicity in MSC are scarce. Significant cytotoxicity of CuO NP was observed in human MSC [48]. In another study, DNA damage and ROS production were detected in rat bone marrow MSC upon treatment with CuO NP with different surface chemistry [49]. Negative impacts of Cu were demonstrated in a report that showed excess Cu in a culture medium to affect MSC proliferation and increase differentiation to bone and adipose tissue [50]. In mouse bone marrow, MSC ZnO NP induced toxicity via mechanisms that involved ROS generation [51]. Genotoxicity of these NP was also found in human MSC after long-term and repetitive exposure [52].

In our study, we intended to compare the biological effects of three types of metal NP. The toxicity tests that we conducted were selected to cover the key mechanisms that NP are believed to affect. We first investigated intracellular ROS production, focusing on a wide range of exposure time intervals (15 min–48 h). We expected ROS to be generated shortly after the application of NP. However, increased ROS production was mostly linked to longer (24 h and 48 h) exposure periods after CuO NP treatment. Upon generation, ROS are scavenged by cellular antioxidant mechanisms that may be overloaded in the case of excessive ROS production. We speculate that increased ROS levels after 24 h and 48 h CuO NP exposure may result from the insufficient activity of antioxidant mechanisms. Furthermore, our CuO NP DLS data indicate the existence of larger NP aggregates after 4 h exposure and the formation of smaller NP clusters (and even expected dissolution of CuO NP) after the longer treatment periods. A similar, but less pronounced, trend was observed for ZnO NP. The smaller NP structures enter the cells more easily and significantly contribute to ROS formation. Therefore, in our experimental system, CuO NP seems to be the agent with the strongest pro-oxidant properties.

Oxidative stress resulting from the accumulation of ROS causes oxidative damage to macromolecules. To further confirm the pro-oxidant activities of the tested metal NP, we looked at markers of oxidative DNA damage and lipid peroxidation. The levels of oxidized DNA bases detected as Fpg-sensitive sites (oxidized purines) revealed relatively modest effects of NP on oxidative DNA damage, which seems to be in line with our data on ROS production. A limited number of significant results was found after the extended Ag NP and shorter CuO NP treatment. Regardless of the activities of antioxidant mechanisms, the lack of significant data may suggest the induction of repair systems that removed the oxidized bases from DNA. Interestingly, in contrast to these results, lipid peroxidation, detected as IsoP levels, was elevated after most of the exposure conditions. This can be partly explained by the fact that no active repair processes exist for damaged lipids that thus accumulate after being attacked by ROS. In this regard, the pro-oxidant properties of the tested NP seem to be rather comparable, although CuO NP induced the most pronounced response. However, considering our data on ROS production, increased IsoP levels should be detected for rather limited exposure conditions, particularly with no effects of ZnO NP treatment. These seemingly inconsistent results can be explained by the fact that we assessed intracellular ROS production, while IsoP can be formed not only intracellularly but also on plasma membrane lipids as a result of an extracellular ROS attack [53]. Hence, our data suggest that all the tested NP induce ROS formation in a culture media resulting in the oxidation of plasma membrane lipids. NP dissolution in a culture media and the presence of Zn^2+^ and Cu^2+^ ions may further contribute to extracellular ROS generation.

DNA strand breaks may originate from the attack of ROS, or the physical interaction of NP with nucleic acids. We looked at both single-strand (ss) and double-strand (ds) DNA break formation. The Comet assay which detects both the ssDNA and dsDNA breaks revealed significant changes, particularly after the Ag NP and CuO NP exposure. The effects of ZnO NP were minimal; this result might be linked with the possible protective role of Zn against oxidative stress [54]. In contrast, micronuclei formation, which corresponds to dsDNA breaks, was very limited in some significant data for the highest tested doses of NP. These observations suggest that NP mostly induced ssDNA breaks, which are probably only partly induced by intracellular ROS generation, therefore likely reflecting the physical interaction of Ag and CuO NP with DNA. The significant decrease of the MN frequency observed after the shorter (24 h) MSC exposure to CuO and Ag NP can be attributed to the increased number of apoptotic cells (the sub-G1 phase cells) at this time point, resulting in the reduced number of live cells in which the MN frequency could be assessed.

Apoptosis induction may be used as a non-specific parameter that reflects the presence of damaged cells. Bax and Bcl-2 are proteins involved in the apoptosis regulation downstream of p53. While Bax expression has a pro-apoptotic effect, Bcl-2 blocks cell death signaling. Their ratio is considered a parameter to show the sensitivity of the cells to apoptotic stimuli [28]. Our results indicate rather consistent effects of the tested NP treatment on pro-apoptotic response, mostly observed for all the experimental conditions except the lowest doses of NP, although there is a trend of more pronounced response for Ag NP and the weaker effects in ZnO NP exposed cells.

Finally, we analyzed the changes in cell cycle phase distribution following metal NP exposure. Our results indicate that most of the cell cycle disturbances appeared after 24 h incubation, while the longest exposure period was linked with the weakest impacts. In line with the results for other parameters, the effects of ZnO NP exposure were comparably the least. The changes detected after 24 h exposure were mostly linked with the decreased proportion of cells in the G1 and S phase and, particularly for Ag NP treated cells, the elevated proportion of cells in the sub-G1 phase, representing apoptotic cells [55]. Thus, NP exposure seemed to affect cell division and the induction of apoptosis after 24 h incubation.

As our results indicate relatively low toxicity of ZnO NP in MSC, these NP might represent a potentially best candidate for a combined NP-stem cell wound treatment. Recent studies suggested that a combination of ZnO NP with natural polymers (cellulose, chitosan, and alginate polymers) might serve as an alternative way to increase the mechanical and antibacterial properties of wound-healing scaffolds [56]. The application of MSC might further improve the healing potential of this approach. It is, however, crucial to exclude any potential negative effect mediated by metal NP in MSC, as detected, e.g., in our recent study [29]. We analyzed the impact of these NP on the expression of phenotypic markers, differentiation potential, expression of genes for immunomodulatory molecules, and production of cytokines and growth factors and found significant changes in MSC differentiation, expression of *FasL*, *Cox-2*, and *Ido*, as well as production of IGF-1 and HGF. Thus, further research is needed to confirm the suitability of metal NP for applications in wound healing.

## 5. Conclusions

Our study is the first investigation in which comprehensive data on the toxicity of several antimicrobial metal NP in mouse MSC are reported. Our results indicate the negative biological impacts of these NP on MSC. The effects depend on the specific NP and exposure conditions: in our experimental setup, ZnO NP had the least adverse impacts on MSC, while CuO NP tended to be the most toxic compound. The tested NP induced intracellular ROS generation, which was most likely partly eliminated by the antioxidant mechanisms, resulting in limited effects on oxidative DNA damage. Lipid peroxidation seemed to be induced in an alternative way, most likely by extracellular ROS. NP predominantly caused ssDNA breaks, in which mechanisms other than ROS-mediated, possibly associated with ions formation, were involved. While all the tested compounds increased the sensitivity of MSC to apoptotic signals, apoptosis was induced only after the Ag NP exposure. The tested NP affected the G1 and S phases of the cell cycle after 24 h exposure.

In summary, the metal antimicrobial NP tested in our study exert a negative response in MSC and might not be optimal for combined wound treatment with MSC. Therefore, a beneficial approach for future combined applications should include more experiments, including thorough NP characterization, preferably in vivo in experimental animals, to confirm these in vitro data. For the potential future applications in humans, a dedicated study should be conducted in which toxicity and biological impacts of metal NP in MSC of human origin will be tested.

## Figures and Tables

**Table 1:** The primer sequences.

Gene	Forward Primer	Reverse Primer
*Bax*	GTGAGCGGCTGCTTGTCT	GGTCCCGAAGTAGGAGAGGA
*Bcl-2*	AGTACCTGAACCGGCATCTG	GGGGCCATATAGTTCCACAAA
*GAPDH*	AGAACATCATCCCTGCATCC	ACATTGGGGGTAGGAACAC

**Table 2:** Average hydrodynamic diameter (z-average) and polydispersity index (PDI) of Ag NP. Green–Yellow color scale is used at all time points for each concentration separately (green > yellow). Values in red indicate conditions for which PDI > 0.7 (see text for details). N/A—not applicable.

	T0		T4		T24		T48	
	Z-Average (d.nm)	SD	Z-Average (d.nm)	SD	Z-Average (d.nm)	SD	Z-Average (d.nm)	SD
Ag NP batch	187.02	16.64	N/A	N/A	N/A	N/A	N/A	N/A
Ag NP 1.5 µg/mL (DMEM + 10% FBS)	86.45	55.57	93.99	45.77	64.30	36.63	61.24	53.07
Ag NP 3.125 µg/mL (DMEM + 10% FBS)	115.94	37.37	122.20	18.28	111.87	47.83	101.14	34.47
Ag NP 6.25 µg/mL (DMEM + 10% FBS)	147.07	22.22	166.60	16.01	176.28	32.43	252.58	33.61
								
	**T0**		**T4**		**T24**		**T48**	
	**PDI**	**SD**	**PDI**	**SD**	**PDI**	**SD**	**PDI**	**SD**
Ag NP batch	0.47	0.06	N/A	N/A	N/A	N/A	N/A	N/A
Ag NP 1.5 µg/mL (DMEM + 10% FBS)	0.66	0.23	0.60	0.18	0.64	0.17	0.63	0.15
Ag NP 3.125 µg/mL (DMEM + 10% FBS)	0.60	0.16	0.71	0.11	0.70	0.16	0.70	0.15
Ag NP 6.25 µg/mL (DMEM + 10% FBS)	0.61	0.14	0.64	0.10	0.59	0.17	0.69	0.17

**Table 3:** Average hydrodynamic diameter (z-average) and polydispersity index (PDI) of ZnO NP. Green –Yellow color scale is used within all time points for each concentration separately (green > yellow). N/A—not applicable.

	T0		T4		T24		T48	
	Z-Average (d.nm)	SD	Z-Average (d.nm)	SD	Z-Average (d.nm)	SD	Z-Average (d.nm)	SD
ZnO NP batch	329.11	41.50	N/A	N/A	N/A	N/A	N/A	N/A
ZnO NP 0.75 µg/mL (DMEM + 10% FBS)	20.36	0.32	19.80	0.18	18.10	0.34	15.87	0.18
ZnO NP 1.5 µg/mL (DMEM + 10% FBS)	23.40	1.11	20.26	0.47	17.73	0.53	15.81	0.40
ZnO NP 3.125 µg/mL (DMEM + 10% FBS)	20.72	0.53	20.71	0.48	17.89	0.43	15.86	0.31
								
	**T0**		**T4**		**T24**		**T48**	
	**PDI**	**SD**	**PDI**	**SD**	**PDI**	**SD**	**PDI**	**SD**
ZnO NP batch	0.12	0.03	N/A	N/A	N/A	N/A	N/A	N/A
ZnO NP 0.75 µg/mL (DMEM + 10% FBS)	0.52	0.01	0.50	0.00	0.47	0.02	0.45	0.01
ZnO NP 1.5 µg/mL (DMEM + 10% FBS)	0.51	0.04	0.50	0.02	0.48	0.01	0.44	0.02
ZnO NP 3.125 µg/mL (DMEM + 10% FBS)	0.51	0.01	0.51	0.03	0.48	0.02	0.44	0.01

**Table 4:** Average hydrodynamic diameter (z-average) and polydispersity index (PDI) of CuO NP. Green–Yellow color scale is used within all time points for each concentration separately (green > yellow). Values in red indicate conditions for which PDI > 0.7 (see text for details). N/A—not applicable.

	T0		T4		T24		T48	
	Z-Average (d.nm)	SD	Z-Average (d.nm)	SD	Z-Average (d.nm)	SD	Z-Average (d.nm)	SD
CuO NP batch	340.99	5.21	N/A	N/A	N/A	N/A	N/A	N/A
CuO NP 0.2 µg/mL (DMEM + 10% FBS)	26.76	7.62	22.91	0.58	19.40	0.38	17.88	0.42
CuO NP 0.3 µg/mL (DMEM + 10% FBS)	48.27	4.76	24.32	0.97	19.79	0.30	18.58	0.16
CuO NP 0.4 µg/mL (DMEM + 10% FBS)	36.01	15.45	27.92	6.39	26.47	3.16	18.56	0.37
								
	**T0**		**T4**		**T24**		**T48**	
	**PDI**	**SD**	**PDI**	**SD**	**PDI**	**SD**	**PDI**	**SD**
CuO NP batch	0.22	0.02	N/A	N/A	N/A	N/A	N/A	N/A
CuO NP 0.2 µg/mL (DMEM + 10% FBS)	0.58	0.14	0.61	0.08	0.50	0.01	0.48	0.02
CuO NP 0.3 µg/mL (DMEM + 10% FBS)	0.66	0.10	0.71	0.02	0.51	0.03	0.48	0.00
CuO NP 0.4 µg/mL (DMEM + 10% FBS)	0.69	0.26	0.71	0.15	0.77	0.04	0.48	0.01

## Data Availability

The data presented in this study are available on request from the corresponding author.

## Supplementary Materials

The following supporting information can be downloaded at https://www.mdpi.com/article/10.3390/toxics11030253/s1, Figure S1: ROS; Figure S2: TEM; Data are contained within the article and Supplementary material.

## Abbreviations

DLS	dynamic light scattering
IsoP	15-F2t-isoprostane
MSC	mesenchymal stem cells
NP	nanoparticle(s)
ROS	reactive oxygen species
TEM	transmission electron microscopy
