# Supplementary material for: Metal Nanoparticles with Antimicrobial Properties: The Toxicity Response in Mouse Mesenchymal Stem Cells
Source: Toxics. 2023 Mar 9;11(3):253. doi: 10.3390/toxics11030253 (PMC10057305; doi:10.3390/toxics11030253)
Supplement: Supplementary file 1 [file toxics-11-00253-s001.zip › toxics-2236701-SI.pdf]

toxics

Metal Nanoparticles with Antimicrobial Properties: The Toxicity Response in Mouse Mesenchymal Stem Cells

Pavel Rossner Jr. [1,†,*], Tereza Cervena [1,2,†], Barbora Echalar [1], Katerina Palacka [1], Alena Milcova [2], Zuzana Novakova [1], Michal Sima [1], Zuzana Simova [1], Jolana Vankova [1] and Vladimir Holan [1].

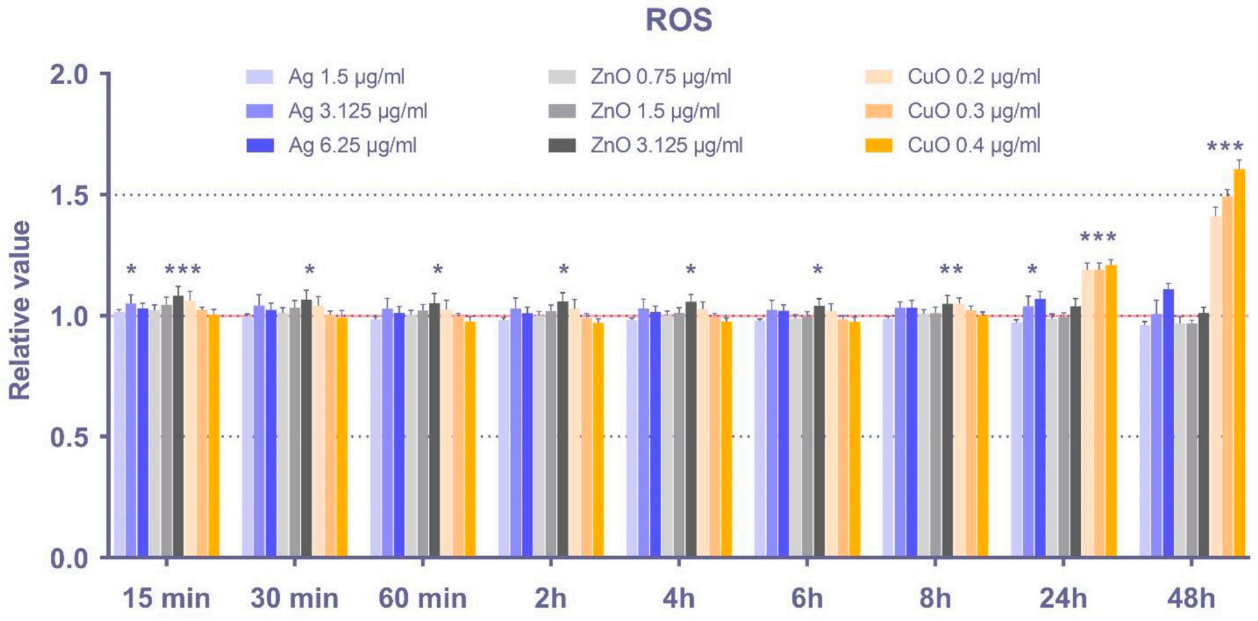

Figure S1. Relative intracellular ROS levels, calculated as ratio between the exposed and control samples, in MSC after 15 min, 30 min, 60 min, 2h, 4h, 6h, 8h, 24h and 48h exposure to selected doses of Ag, ZnO and CuO NP. The average values from six independent experiments are presented. The red line indicates the reference ROS levels in the controls. The asterisk denotes significant changes when compared with the untreated controls ($p < 0.05$).

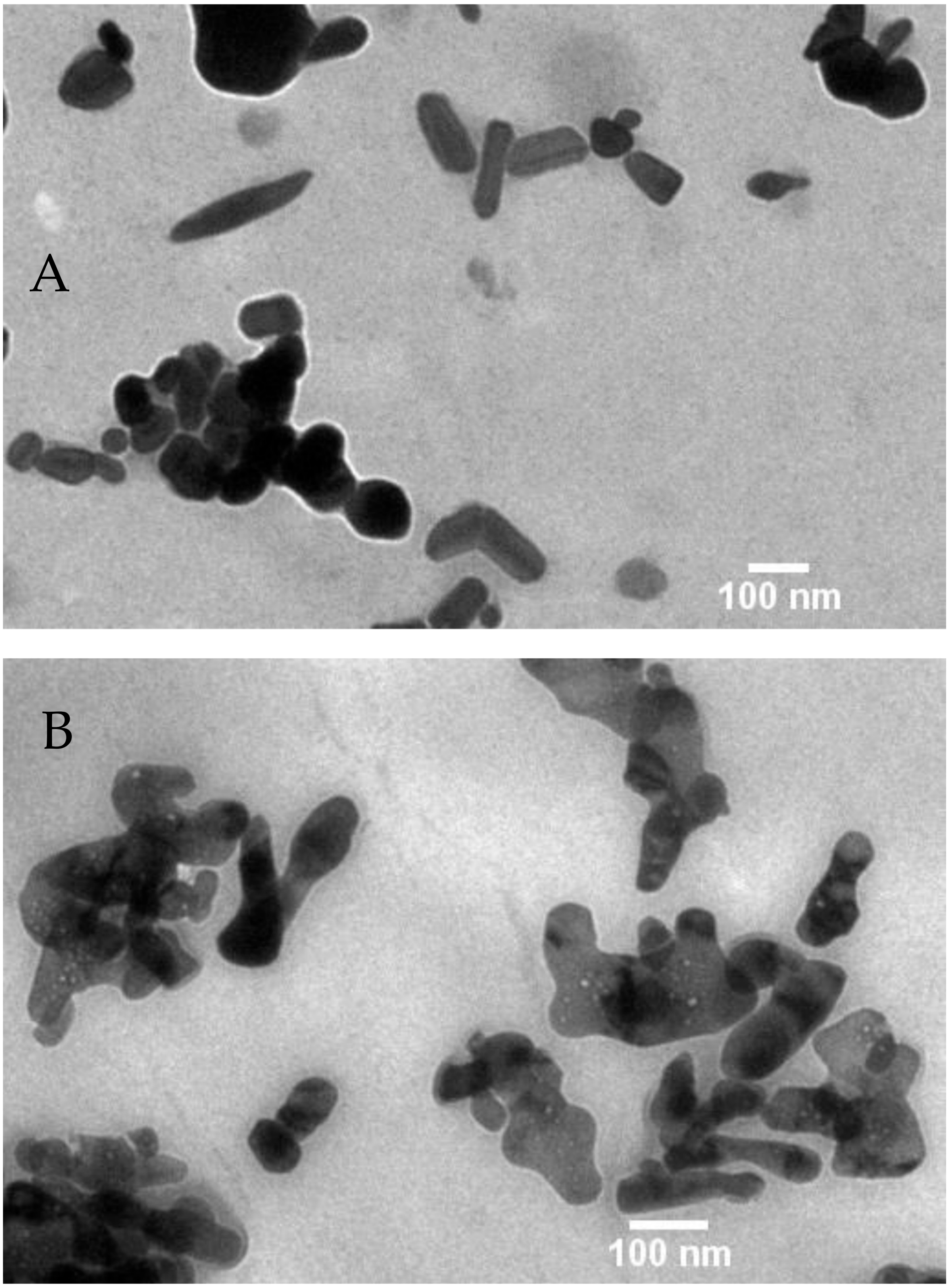

Figure S2. Transmission electron microscopy images of batch ZnO (A) and CuO (B) at concentration 2.56 mg/ml.
